# A Heterologous Multiepitope DNA Prime/Recombinant Protein Boost Immunisation Strategy for the Development of an Antiserum against *Micrurus corallinus* (Coral Snake) Venom

**DOI:** 10.1371/journal.pntd.0004484

**Published:** 2016-03-03

**Authors:** Henrique Roman Ramos, Inácio de Loiola M. Junqueira-de-Azevedo, Juliana Branco Novo, Karen Castro, Clara Guerra Duarte, Ricardo A. Machado-de-Ávila, Carlos Chavez-Olortegui, Paulo Lee Ho

**Affiliations:** 1 Centro de Biotecnologia, Instituto Butantan, São Paulo, São Paulo, Brazil; 2 Centro de Toxinologia Aplicada, Instituto Butantan, São Paulo, São Paulo, Brazil; 3 Departamento de Bioquímica, Universidade Federal de Minas Gerais (UFMG), Belo Horizonte, Minas Gerais, Brazil; 4 Unidade Acadêmica de Ciências de Saúde, UNESC, Criciúma, Santa Catarina, Brazil; Liverpool School of Tropical Medicine, UNITED KINGDOM

## Abstract

**Background:**

Envenoming by coral snakes (Elapidae: *Micrurus*), although not abundant, represent a serious health threat in the Americas, especially because antivenoms are scarce. The development of adequate amounts of antielapidic serum for the treatment of accidents caused by snakes like *Micrurus corallinus* is a challenging task due to characteristics such as low venom yield, fossorial habit, relatively small sizes and ophiophagous diet. These features make it difficult to capture and keep these snakes in captivity for venom collection. Furthermore, there are reports of antivenom scarcity in USA, leading to an increase in morbidity and mortality, with patients needing to be intubated and ventilated while the toxin wears off. The development of an alternative method for the production of an antielapidic serum, with no need for snake collection and maintenance in captivity, would be a plausible solution for the antielapidic serum shortage.

**Methods and Findings:**

In this work we describe the mapping, by the SPOT-synthesis technique, of potential B-cell epitopes from five putative toxins from *M*. *corallinus*, which were used to design two multiepitope DNA strings for the genetic immunisation of female BALB/c mice. Results demonstrate that sera obtained from animals that were genetically immunised with these multiepitope constructs, followed by booster doses of recombinant proteins lead to a 60% survival in a lethal dose neutralisation assay.

**Conclusion:**

Here we describe that the genetic immunisation with a synthetic multiepitope gene followed by booster doses with recombinant protein is a promising approach to develop an alternative antielapidic serum against *M*. *corallinus* venom without the need of collection and the very challenging maintenance of these snakes in captivity.

## Author Summary

Coral snakes are a group of deadly venomous snakes that exhibit a characteristic red, yellow/white, and black coloured banding pattern. Accidents involving these snakes tend to be very severe or even lethal, causing peripheral nervous system depression with muscle paralysis and vasomotor instability. The only acceptable medical treatment for snakebite accidents is the administration of an antivenom, generally produced by immunising horses with the snake venom. Nonetheless, for what concerns the antielapidic serum production in Brazil, the total amount of venom available for horse immunisations is insufficient. This is mainly due to the small size of coral snake glands, their underground life style, combined with its very low survival rates in captivity. Moreover, cases of patients being intubated and ventilated as a consequence of antivenom shortage in USA have also been registered. In this work, we present an alternative method for the development of antielapidic serum, which does not rely upon snake capture. This serum was produced by a heterologous DNA prime—with a multiepitope DNA string coding for the most reactive epitopes from the most abundant toxins of *M*. *corallinus*, a coral snake which occupy highly populated areas in Brazil—followed by recombinant multiepitope protein boost immunisation of mice.

## Introduction

Envenomation by snakebite is a common and generally harmful, environmental and occupational neglected tropical disease that constitutes a highly relevant public health problem with worldwide mortality estimated to be around 50,000 deaths annually [1]. In the Americas, although most of the registered cases of snake envenomation are due to snakes from the Viperidae family [2], accidents caused by members of the Elapidae family can also be severe or even lethal [3]. Distributed throughout the tropical and subtropical regions around the world, the Elapidae family consists of 325 species divided into 61 genera of potentially deadly neurotoxic venomous snakes that exhibit a wide range of sizes and are characterised by hollow and proteroglyphous fixed fangs through which venom is injected.

The coral snakes are the only elapids found in the New World, being *Micrurus* the most diverse and abundant genus across Americas [4]. In Brazil, the envenomation accidents reported are mainly due to *M*. *corallinus* and *M*. *frontalis*, which occupy highly populated areas in central, south and southeast of the country [5]. For this reason, the immunisation of horses with equal amounts of *M*. *corallinus* and *M*. *frontalis* venoms is used at Butantan Institute for the production of the Brazilian coral snake antivenom [6], which is the only accepted medical treatment for coral snakebite envenomation [7].

*Micrurus spp*. coral snakes have an average dry venom yield of 13.87 mg [8], which results in the need of snake collections composed of numerous specimens in order to obtain sufficient amounts of venom for horse immunisation. On the other hand, due to characteristics such as fossorial habit, relatively small sizes and ophiophagous diet, it is very challenging to capture and keep these snakes in captivity, as survival rate rarely exceeds one year [9]. These limitations in maintenance, the small size of their venom glands and, consequently, the low yield of venom, have been the major factors jeopardising the production of the Brazilian antielapidic serum.

Additionally, being snakebite a health problem that mainly afflicts the poorest regions of the world [10], antivenom production holds very limited commercial value, which not only hinders its production by major pharmaceutical companies but also results in an increased shortage of antivenom. As a matter of fact, since 2003, Pfizer/Wyeth, discontinued the manufacture of ANTIVENIN^®^, the only FDA-approved coral snake antivenom used for the treatment of accidents caused by *Micrurus fulvius*, a coral snake found in the southeastern USA. Furthermore, since 2008, all of the 2003 antivenom lots have expired, culminating in critical situations of patients being intubated and ventilated while toxins wear off. Under these circumstances, there is an increase not only in the morbidity of these accidents, but also of registered cases of people dying as a result of antivenom shortage [11–13].

Another issue that should be addressed is that the venom glands of snakes produce a variety of proteins and biologically active peptides with only a small percentage of those molecules being actually responsible for the biological manifestations observed after envenomation. As a result, antivenoms contains antibodies against an extensive number of different proteins, irrespective of their toxicity or immunogenicity, leading to a reduction in the antivenom’s efficacy and to an increased probability of developing serum sickness reactions due to large volumes of equine proteins [14]. The development of an alternative, but still efficient immunising protocol for the generation of coral snake antivenom, with less reliance upon snake collection/maintenance and composed solely of toxin-specific antibodies, would, therefore, be ideal for the treatment of envenomation by coral snake bites.

The use of recombinant coral snake toxins as immunogens would be a reasonable way to accomplish both issues but, although these molecules did induce an immune response that indicated the recognition of the native proteins, very complicated steps were required for protein refolding [15]. On the other hand, however, the use of DNA immunisation to evoke IgG antibody titres and protective responses for the production of snake antisera have also been described [16–19]. Furthermore, researchers from the Liverpool School of Tropical Medicine, UK, demonstrated that the genetic immunisation of mice with a multiepitope DNA string coding for the most antigenic epitopes of metalloproteinases from *Echis ocellatus* (an African viper) could be used for the generation of an antiserum that neutralised the toxicity of different African snakes [20], similar to the responses observed when rabbits were immunised with recombinant toxins [21]. These observations not only indicate that the DNA immunisation is a plausible way of developing specific and neutralising antibodies against snake venoms with no need for recombinant protein expression and purification from heterologous organisms such as *Escherichia coli*, but alto indicates that these neutralising antisera could be developed by the genetic immunisation of animals with the most antigenic epitopes, only.

In a previous work, after the transcriptomic analysis of *M*. *corallinus* venom gland, the predominant proteins in the venom were identified and five toxins that could represent good antigenic candidates were chosen for DNA immunisations, providing an initial evidence of the feasibility of this approach for an antielapidic sera development [22]. Among the proposed candidates, there are four three-fingered toxins (3FTx) and one putative *M*. *corallinus* phospholipase A_2_, which were selected based on the abundance of each transcript.

The first antigen selected (Ag1) is a 3FTx similar to a previously characterised as neurotoxin homolog 8 (Nxh8), which differs from most 3FTx as it shows an extra disulphide bond in the first loop [23]. The second one (Ag2) refers to a more typical 3FTx and is homologous to the previously described Nxh7, Nxh3 and Nxh1 neurotoxins [24]. The other two 3FTx (Ag3 and Ag4) represent new identified proteins with similarity of no more than 50% to the sequences of 3FTx in the databanks. The fifth selected antigen candidate (Ag5) corresponds to the putative *M*. *corallinus* phospholipase A_2_ (PLA_2_).

In this work we describe the mapping, by the SPOT-synthesis technique [25], of potential B-cell epitopes from these five putative toxins. These epitopes were then analysed through different *in silico* methods and used for the design of two multiepitope DNA strings for the genetic immunisation of female BALB/c mice. By the end of the immunisation period, animals were bled and sera were subjected to further analysis concerning its neutralisation capabilities.

## Materials and Methods

### Peptide synthesis on cellulose membranes

**Fig 1:**
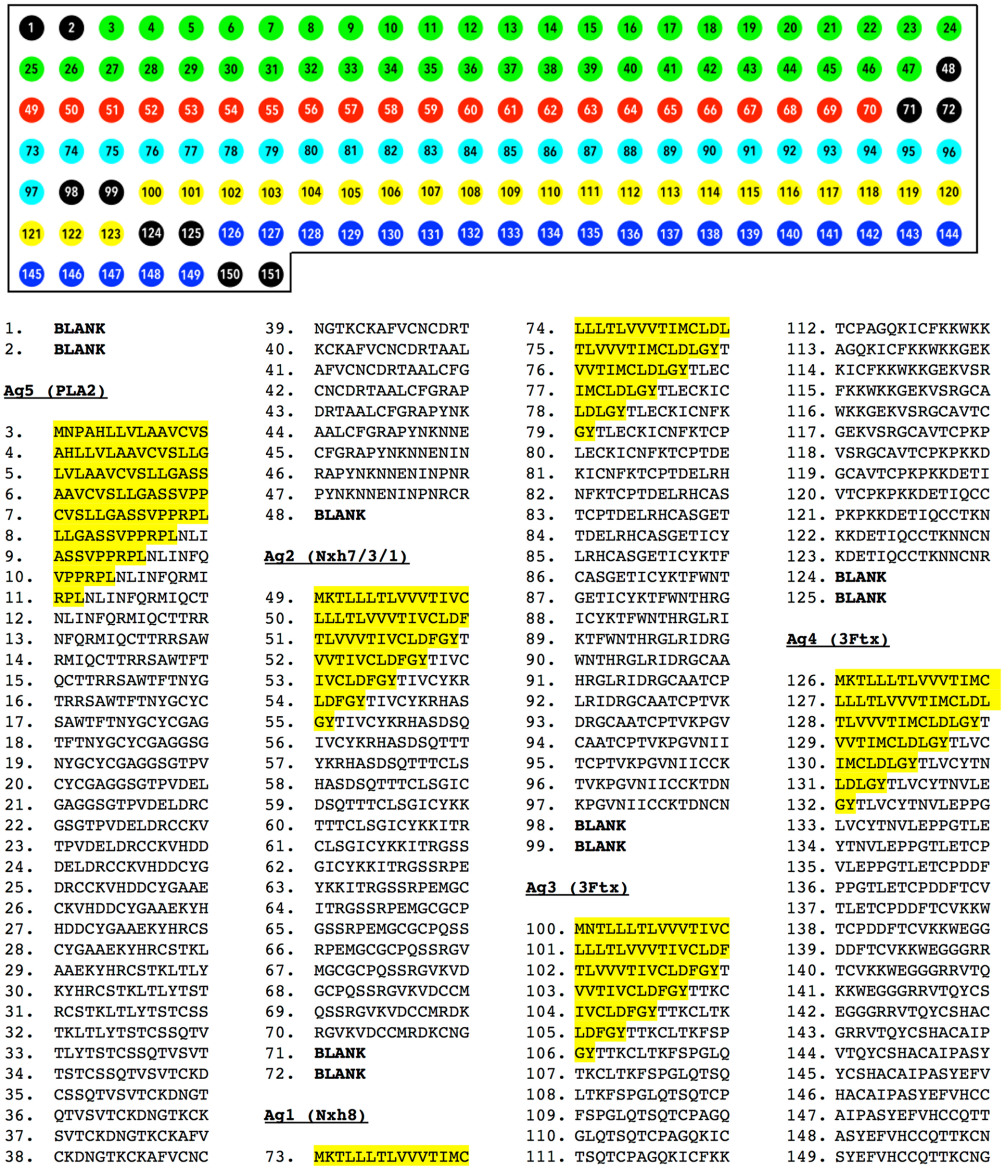
SPOT peptide synthesis scheme. (Black circles) Blank spots. (Cyan circles) Spots from Ag1 (Nxh8— 3FTx). (Red Circles) Spots from Ag2 (Nxh 7/3/1— 3FTx). (Yellow circles) Spots from Ag3 (3Ftx). (Blue circles) Spots from Ag4 (3Ftx). (Green circles) Spots from Ag5 (PLA_2_). (Highlighted spot sequences) Signal peptides, which were not considered for multiepitope gene design.

The identification of potential B-cell epitopes from the five most abundant toxins that constitute the venom of *Micrurus corallinus* [22] was performed by the SPOT-synthesis technique [25]. For this procedure, overlapping pentadecapeptides, frameshifted by three residues and spanning the whole sequences of all these toxins were adsorbed into a cellulose membrane according to the protocol of Laune et al. [26]. The cellulose membranes were obtained from Intavis (Koln, Germany); fluorenylmethyloxycarbonyl amino acids and N-Ethyl(hydroximino)cyanoacetate were from Novabiochem. A ResPep SL/AutoSpot SL Automatic Spot synthesiser (IntavisAG, Bioanalytical Instruments, Germany) was used for the automated peptide synthesis in the membrane. After assembling the peptide sequences, the side-chain protecting groups were removed by treatment with trifluoroacetic acid. A membrane map of epitopes can be found in Fig 1.

### Immunoassay and regeneration

For the identification of immunoreactive peptides, after an overnight blocking step with 3% bovine serum albumin (BSA) diluted in phosphate buffered saline with 0.05% (v/v) Tween-20 (PBS-T), the SPOT membrane was probed with a 1:1000 dilution of a monospecific anti-*M*. *corallinus* horse antiserum (whole IgG, kindly provided by the antivenom facility of Butantan Institute, São Paulo, Brazil). Antibody binding was detected with an alkaline phosphatase-conjugated anti-horse IgG (Sigma Aldrich) and detection was performed with 60 μL of MTT 0.12M (methylthiazolyldiphenyl-tetrazolium bromide, Sigma Aldrich), 50 μL BCIP 0.16M (5-bromo-4-chloro-3-indolyl phosphate, Sigma Aldrich) and 40 μL MgCl_2_ 1M diluted in 10mL of citrate buffered saline, pH 7.0 (137 mM NaCl, 3 mM KCl, 10mM citric acid). The membrane was digitalised using a colour scanner (ScanJet 3670, Hewlett-Packard) and subjected to densitometric intensity analysis with ImageJ—image processing software [27]. In order to eliminate unspecific binding of secondary antibody to spots, the membrane was also probed and detected with secondary antibody alone. A spot was considered immunoreactive when the relative density value obtained after incubation with both primary and secondary antibodies was higher than the value obtained by the incubation with the secondary antibody, alone.

### Multiepitope DNA strings design and synthesis

Two multiepitope DNA strings were designed based on the results obtained with the SPOT-synthesis technique [25]. One of them codes for the reactive epitopes associated with the four selected 3FTx while the other one codes for the reactive epitopes associated with the putative phospholipase A_2_ (PLA_2_) toxin. All epitopes sequences were separated by a six amino acid residues linker and all cysteine codons were exchanged by serine codons in order to avoid the formation of disulphide bond-mediated protein multimerisation. The codon usage was optimised for both *Mus musculus* and *Escherichia coli* expression according to the *Codon Usage Database* (*Kazusa DNA Research Institute*) [28]. To facilitate further molecular cloning into expression vectors, a *Xho*I and a *Sfi*I restriction sites were inserted at the 5’ region of each DNA string, while a *Pst*I restriction site was inserted at the 3’ region of each DNA string. Theses DNA strings were synthesised by GeneArt^®^ Gene Synthesis (Thermo Fischer Scientific).

### 3D –modelling

The three-dimensional structure of all the five toxins described in this work have not been resolved yet. However, in order to obtain the approximated spatial localisation of reactive epitopes, we performed some protein structure homology modelling using the SWISS-MODEL workspace tool for molecular modelling [29–31]. Ramachandran plots [32] and QMEAN [33] scores were used for quality assessment and models were selected based on their Global Model Quality Estimation (GMQE) values [31]. Briefly, the Ag1 (Nxh8) homology model was obtained based on the crystal structure (PDB ID: 3nds) of *Naja nigricollis* toxin alpha (GMQE: 0.80 / Seq. identity: 54.10 / Seq. similarity: 0.48). The Ag2 (Nxh7/3/1) homology model was obtained based on the NMR structure (PDB ID: 1nor) of neurotoxin II from *Naja naja oxiana* (GMQE: 0.78 / Seq. identity: 40.35 / Seq. similarity: 0.48). The Ag3 (3FTx) homology model was obtained based on the crystal structure (PDB ID: 2h8u) of Bucain, a cardiotoxin from the Malayan Krait *Bungarus candidus* (GMQE: 0.89 / Seq. identity: 57.63 / Seq. similarity: 0.51). The Ag4 (3FTx) homology model was obtained based on the crystal structure (PDB ID: 4iye) of the green mamba, *Dendroaspis angusticeps*, ρ-Da1a toxin (GMQE: 0.75 / Seq. identity: 40.00 / Seq. similarity: 0.40). And, finally, the Ag5 (PLA_2_) homology model was obtained based on the crystal structure (PDB ID: 1yxh) of a phospholipase A_2_ from *Naja naja sagittifera* (GMQE: 0.82 / Seq. identity: 58.97 / Seq. similarity: 0.50). Protein images were generated using DeepView (Swiss PDB Viewer) [34].

### Hydrophobicity and antigenic index analysis

We also decided to evaluate the hydropathic profile and antigenic index of all antigens so we could analyse if the reactive epitopes are localised in highly hydrophilic and antigenic regions of their respective proteins. This would corroborate and reinforce the empirical results obtained by the SPOT-synthesis technique. The hydropathic characters of all proteins were evaluated using the Kate & Doolittle algorithm [35] while the antigenic index were computed using the Jameson & Wolf algorithm [36]. All images were created with Protean computer program (DNASTAR Inc. Madison, Wisconsin, USA).

### PCR and molecular cloning of the complete coding sequences of selected antigens

The complete coding sequences corresponding to the mature portions of the five selected antigens were PCR amplified from a previously constructed cDNA library [22]. Both *Kpn*I and *Xho*I restriction sites were included in the 5’ end of the forward primer while a *Not*I restriction site was included in the 5’ end of the reverse primer. Each one of the amplicons were cloned into the *Kpn*I and *Not*I endonucleases sites of individual pSECTAG2A mammalian expression vectors (*Thermo Fischer Scientific)*. To avoid the expression of the c-myc epitope located at the 3’ region of the vector multi cloning site, a stop codon was introduced in the reverse primer. Alternatively, for the heterologous recombinant expression of toxins in *Escherichia coli* cells, the amplicons were also cloned into the *Xho*I and *Not*I restriction sites of a high-copy T7 promoter-based *E*. *coli* expression vector (pAE) [37].

### Molecular cloning of the multiepitope DNA strings

Both multiepitope DNA strings were cloned either into the *Sfi*I and *Pst*I restriction sites of pSECTAG2A plasmids (for the genetic immunisations protocols) or into the *Xho*I and *Pst*I restriction sites of pAE vectors (for the heterologous recombinant expression in *Escherichia coli* cells).

### Transient transfection in COS-7 cells and RT-PCR

The correct transcription of toxins’ cDNA sequences by mammalian host cells was investigated by transiently transfecting COS-7 cells (ATCC CRL 1651), which were maintained in Dulbecco's Modified Eagle´s Medium (DMEM; Life Technologies, USA) supplemented with 2 mM L-glutamine, 100U⋅mL^-1^ penicillin, 100μg⋅mL^-1^ streptomycin, 0.25μg⋅mL^-1^ amphotericin B (Thermo Fischer Scientific, USA) and 10% foetal bovine serum (FBS; Cultilab, Campinas, SP, Brazil). Individual pSECTAG2A vectors, cloned with the complete cDNA sequences of each toxin [22], were used for the transient transfection of COS-7 cells using Lipofectamine 2000 (Thermo Fischer Scientific, USA) according to the manufacturer's instructions. Cells were washed three times with Phosphate Buffered Saline (PBS) 48 h after transfection and the medium was replaced with DMEM without FBS. After 24 h incubation, both medium and cells were collected—after centrifugation for 10 minutes at 3000 *g—*and stored at -20°C until use.

After transfection, cells had been treated with Trizol (Thermo Fischer Scientific, USA) for the isolation of total mRNA, which were reverse transcribed using an oligo(dT)20 primer (Thermo Fisher Scientific, USA). Total cDNA was than subjected to PCR amplifications for the detection of toxins’ cDNAs.

### Western blot of COS-7 cell extracts

The heterologous toxin expressions by all COS-7 cells previously transfected were assessed by Western Blot analysis of cell extracts. For this, a SDS-PAGE with the cells extracts was performed and the proteins transferred to a nitrocellulose membrane. After an overnight blocking at 4°C with 10% (v/v) non-fat dry milk diluted in PBS-T, membrane was incubated with a 1:3000 dilution of monospecific anti-*Micrurus corallinus* horse antiserum (kindly provided by the antivenom facility of Butantan Institute) for 90 minutes at constant agitation at room temperature. Free, non-bound primary antibodies were removed with three 30 minutes’ washes in PBS-T. Goat anti-horse IgG-HRP antibodies (Sigma Aldrich) were used as secondary antibody at a dilution of 1:5000. Membranes were probed with ECL Prime detection reagent (GE Healthcare) according to manufacturer’s instruction.

### Preparation of immunogens for GeneGun DNA immunisations

Purified pSECTAG2A plasmid (vehicle control), pSECTAG2A-*ag1*, pSECTAG2A-*ag2*, pSECTAG2A-*ag3*, pSECTAG2A-*ag4*, pSECTAG2A-*ag5*, pSECTAG2A-*3ftx*-multiepitope, and pSECTAG2A-*pla2*-multiepitope were precipitated onto 1.6μm gold beads and coated on the inner surface of Tefzel ETFE Fluoropolymer resin tubing according to the manufacturer’s protocol (BioRad Laboratories, Inc.). The final quantity of DNA/gold beads for each shot was adjusted to 1 μg of DNA / 0.5 mg Au.

### Recombinant protein expression and purification

For the recombinant expression of toxins or multiepitope proteins, each one of the pAE plasmid constructions described before were introduced, by heat shock, into chemically competent *Escherichia coli* BL21 (DE3) cells (Thermo Fischer Scientific, USA), which were grown on Luria-Bertani (LB) medium and induced for three hours by the addition of 1mM isopropyl-1-thio-β-D-galactopyranoside (IPTG) when an OD600 (optical density at 600nm) of 0.6 was achieved. After the induction period, cells were collected by centrifugation and mechanically lysed by French Press (Thermo Fischer Scientific, USA). Recombinant proteins were expressed as inclusion bodies and were solubilised with 20 mL of solubilisation buffer (8 M urea, 50 mM Tris-Cl, 5 mM β-mercaptoethanol, pH 7.4).

After complete solubilisation, recombinant proteins were purified by immobilised metal ion affinity chromatography (IMAC). For this procedure, proteins were adsorbed on a 5mL column previously charged with Ni^+2^ and equilibrated with 5 column volumes (CV) of solubilisation buffer without β-mercaptoethanol. Washing procedures were performed with 5 CV of wash buffer (3M urea, 40mM imidazole, 150mM NaCl, 50mM Tris-Cl, pH 7.4). Protein elution were accomplished with 5 CV of elution buffer (3M urea, 1M imidazole, 150 mM NaCl, 50mM Tris-Cl, pH 7.4). Finally, before these expressed proteins could be used in immunisation regimens, imidazole was removed by simple dialysis against PBS buffer containing 3M urea (to avoid protein precipitation). The purity and concentration of recombinant protein were evaluated and quantified by SDS-PAGE densitometry analysis with ImageJ—Image processing software [27].

### Immunisation regimens

#### Genetic immunisation

7 weeks old female Balb/C mice (18–20 g) had their abdomen shaved and were subjected to four high pressure (350 psi) DNA “shots” into the epidermal layer using the Helios GeneGun (Bio-Rad Laboratories, Inc.). Animals were divided groups of five animals, with each group being immunised on weeks 0, 2, 4, 8 and 12 with one of the seven previously described pSECTAG2A constructions (*ag1*, *ag2*, *ag3*, *ag4*, *ag5*, *3ftx* multiepitope or *pla2* multiepitope) or with the control empty pSECTAG2A plasmid, alone. Animals had their blood collected, by retro-orbital bleeding, one week after the last immunisation (week 13).

#### Heterologous DNA prime-recombinant protein boost immunisation

4 weeks after the last genetic immunisation (week 16), all groups of genetically immunised animals were subjected to heterologous booster immunisation regimen with their related recombinant multiepitope antigens. Animals that were immunised with any one of the *3ftx* cDNAs (ags 1 to 4) or with the *3ftx* multiepitope DNA string, received booster doses with the recombinant 3FTx multiepitope protein. On the other hand, animals that were immunised with either the putative pla_2_ cDNA or with the *pla2* multiepitope DNA string, received booster doses of the recombinant PLA_2_ multiepitope protein. The booster regimen consisted of four doses of 10μg of recombinant protein, adsorbed onto 10% (vol./vol.) Alhydrogel [2% Al(OH)_3_] (kindly provided by the Formulation Section of Butantan Institute, São Paulo, Brazil), at weeks 16, 18, 20 and 22. Blood was collected by retro-orbital bleeding, one week after the last immunisation (week 23).

#### Immunisation with recombinant proteins

7 weeks old female Balb/C mice (18–20 g) were immunised with *E*. *coli*-derived recombinant proteins that were expressed and purified as previously described. For this regimen, animals were immunised with five doses of 10μg of recombinant protein, adsorbed onto 10% (vol./vol.) Alhydrogel [2% Al(OH)_3_] (kindly provided by the Formulation Section of Butantan Institute, São Paulo, Brazil), at weeks 0, 2, 4, 6 and 8. Animals had their blood collected, by retro-orbital bleeding, one week after the last immunisation (week 9).

### Enzyme-Linked Immunosorbent Assay (ELISA)

For the determination of the total IgG titre from the different antisera or from antielapidic antivenom produced by Butantan Institute, 96-well microtitre plates were coated with either 100 μL of purified recombinant antigens (10 μg⋅ml^-1^ in Carbonate-Bicarbonate Buffer, pH 9.6) or with 100 μL of *Micrurus corallinus* venom (10 μg⋅ml^-1^ in Carbonate-Bicarbonate Buffer, pH 9.6). After three washes with PBS-T, plates were blocked with 5% non-fat milk/PBS-T (m/V) at 37°C for 1 h. Serial dilutions of each serum in PBS-T were added to the wells and microtitre plates incubated for 1 h at 37°C. Bound antibodies were detected with a 1:5000 dilution of a commercial peroxidase-conjugated anti-mouse IgG (Sigma Aldrich). Detection was performed with 8 mg o-phenylelediamine (OPD) diluted in 20 mL of 0.2 M citrate-phosphate buffer, pH 5.0, in the presence of 10 μL of 30% H_2_O_2_. Reaction was stopped by adding 50 μL of 4M H_2_SO_4_ to each well. Absorbances were measured at 492 nm and titres were determined as the highest dilution, in which an absorbance value ≥ 0.1 was observed. ELISA experiments were performed in simultaneous duplicates with all detection reaction being stopped at the same time.

### Neutralisation of lethal activity

In order to evaluate the neutralisations capabilities of all experimental sera conceived during the immunisations protocols performed in this study, 3LD_50_ (21 μg) [38] of *Micrurus corallinus* venom were diluted in physiological saline to a final volume of 100 μL and mixed with the 100 μL of each serum. All venom/antiserum mixtures were, then incubated at 37°C for a total of 30 min before being intraperitoneally administered to groups of five female Balb/c mice weighting around 20 g. Animals were monitored every 6 hours with the number of deaths being recorded until 48 h after injection. For positive and negative controls groups, 3LD_50_ of venom were also incubated with either 100 μL of Butantan’s antielapidic antivenom, 100 μL of monospecific anti-*M*. *corallinus* horse antiserum or with 100 μL of serum from naïve mice, respectively.

### Ethics statement

All animal experimentation protocols were performed in conformity with the Ethical Principles on Animal Research of the Brazilian College of Animal Experimentation (COBEA) and were previously revised and approved by the Ethics Committee on Animal Research of Butantan Institute under identification number **657/09**.

## Results and Discussion

### Epitope mapping

**Fig 2:**
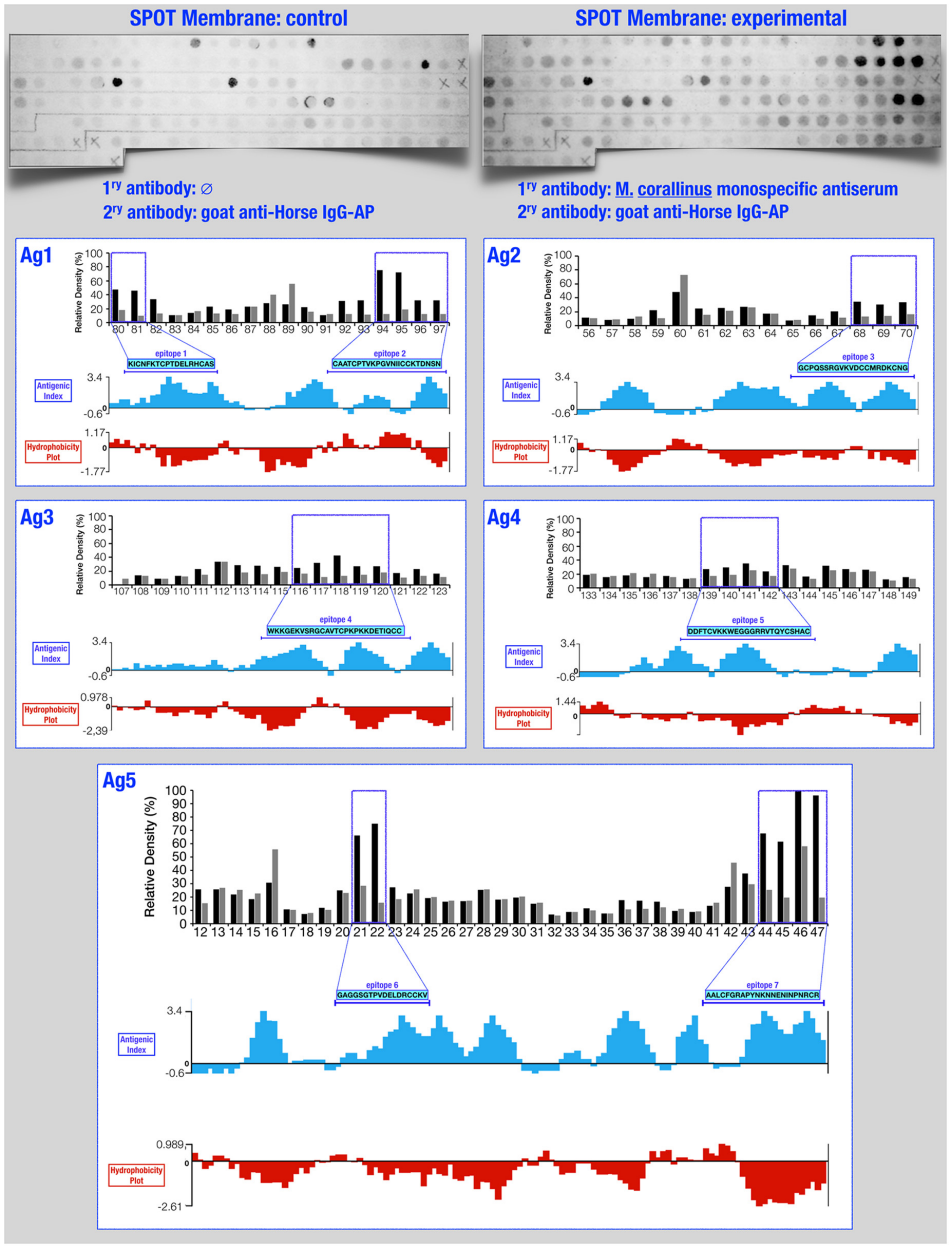
Epitope mapping through the SPOT-synthesis technique. The relative density values of every single spot were determined and plotted into bar graphs. ***Black bars*** correspond to the relative density of each spot after incubation with both primary and secondary antibodies. ***Grey bars*** correspond to the relative density of each spot after incubation with only the secondary antibody (***negative control***). Positive spots are delimited by ***blue rectangles*** and their corresponding amino acid sequences (***highlighted in clear blue rectangles***) are aligned with its respective antigen’s ***Antigenic index*** (Jameson-Wolf) and ***Hydrophobicity plot*** (Kyte-Doolittle). Spots IDs (below bars) are the same as those described in Fig 1.

Two multiepitope DNA-strings were designed by identifying reactive B-cell epitopes in four major three-fingered toxins and one phospholipase A_2_ from *M*. *corallinus* venom. For this, synthetic pentadecapeptides covering the entire amino acid sequences of these toxins were adsorbed on a nitrocellulose membrane (Fig 1), which was incubated with a monospecific anti *M*. *corallinus* horse antiserum and revealed with an alkaline phosphatase-conjugated goat anti-horse IgG as secondary antibody. Unspecific spots were identified by incubating the SPOT membrane with only the secondary antibody. A spot was considered immunoreactive when its relative density value after incubation with both primary and secondary antibodies was higher than its relative density value obtained after incubation with only the secondary antibody (Fig 2).

Represented by spots 80/81 and 94/95/96/97, two linear epitopes with amino acid sequences KICNFKTCPTDELRHCAS (**Epitope 1**) and CAATCPTVKPGVNIICCKTDNSN (**Epitope 2**) were identified in the primary structure of Ag1 (Fig 2—Ag1). Likewise, the densitometric analysis of spots associated with the primary structure of Ag2 showed a single epitope (Fig 2—Ag2) represented by spots 68/69/70 and with amino acid sequence GCPQSSRGVKVDCCMRDKCNG (**Epitope 3**). In the same way, a single 27-mer epitope, represented by spots 116/117/118/119/120 and with amino acid sequence WKKGEKVSRGCAVTCPKPKKDETIQCC (**Epitope 4**) was detected on Ag3 (Fig 2—Ag3) and a single 24-mer epitope, represented by spots 139/140/141/142 and with amino acid sequence DDFTCVKKWEGGGRRVTQYCSHAC (**Epitope 5**) was detected on Ag4 (Fig 2—Ag4). In the case of the putative PLA_2_, two linear epitopes were detected (Fig 2—Ag5), represented by spots 21/22 and 44/45/46/47. The amino acid sequences of these epitopes are, respectively, GAGGSGTPVDELDRCCKV (**Epitope 6**) and AALCFGRAPYNKNNENINPNRCR (**Epitope 7**).

**Fig 3:**
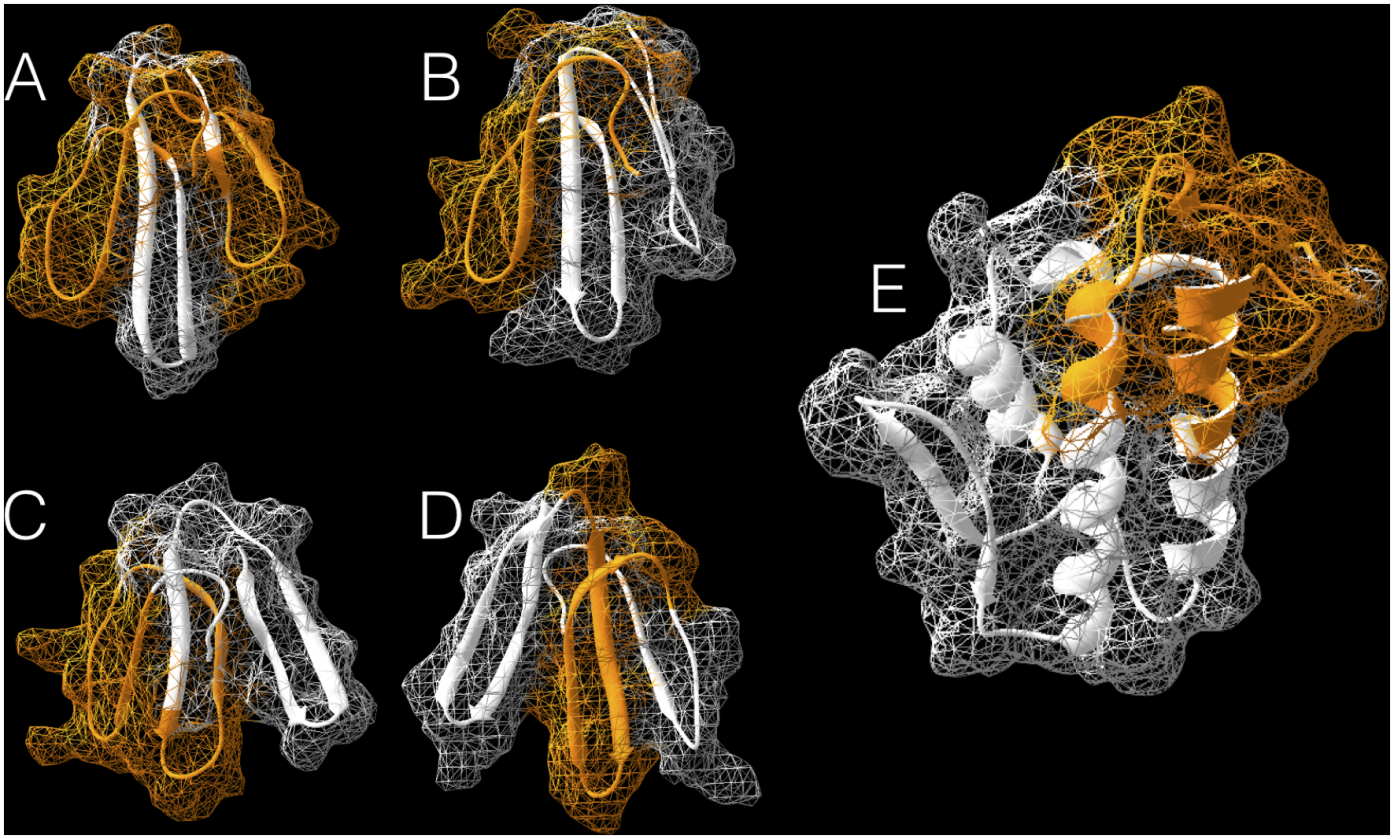
Three dimensional modelling with solvent-accessible surface area (SASA) of all five antigens described in this work. Reactive epitopes are highlighted in orange. (A) 3D model of Ag1 (Nxh8) based on the crystal structure (PDB ID: 3nds) of *Naja nigricollis* toxin alpha (GMQE: 0.80 / Seq. identity: 54.10 / Seq. similarity: 0.48). (B) 3D model of Ag2 (Nxh7/3/1) based on the NMR structure (PDB ID: 1nor) of neurotoxin II from *Naja naja oxiana* (GMQE: 0.78 / Seq. identity: 40.35 / Seq. similarity: 0.48). (C) 3D model of Ag3 (3FTx) based on the crystal structure (PDB ID: 2h8u) of Bucain, a cardiotoxin from the Malayan Krait *Bungarus candidus* (GMQE: 0.89 / Seq. identity: 57.63 / Seq. similarity: 0.51). (D) 3D model of Ag4 (3FTx) based on the crystal structure (PDB ID: 4iye) of the green mamba, *Dendroaspis angusticeps*, ρ-Da1a toxin (GMQE: 0.75 / Seq. identity: 40.00 / Seq. similarity: 0.40). (E) 3D model of Ag5 (PLA_2_) based on the crystal structure (PDB ID: 1yxh) of a phospholipase A_2_ from *Naja naja sagittifera* (GMQE: 0.82 / Seq. identity: 58.97 / Seq. similarity: 0.50). All images were generated using DeepView (Swiss PDB Viewer) [34].

Considering that the hydrophilic regions of a protein are precisely those that, in theory, are more exposed to the immune system and consequently have a higher reactivity when in contact with an anti-*M*. *corallinus* serum, we decided to compare the position of these epitopes within an antigenic index and a hydrophilic profile of their respective antigens. The results clearly demonstrate that all epitopes are positioned within the antigenic and hydrophilic regions (Fig 2—Ags1–5). Additionally, when these epitopes were mapped into the three-dimensional models we created, we could observe that these epitopes are occupying large accessible surface areas (Fig 3), corroborating the empirical results obtained by this epitope mapping technique. Furthermore, concerning the PLA_2_ 3D model, it is also worth noting that despite the two detected epitopes are located in opposite sides in the primary structure of the protein, they are situated in the same spatial region of the protein, which strongly suggests that these peptides are, indeed, important for an effective immune response (Fig 3E).

### Multiepitope DNA strings design

**Fig 4:**
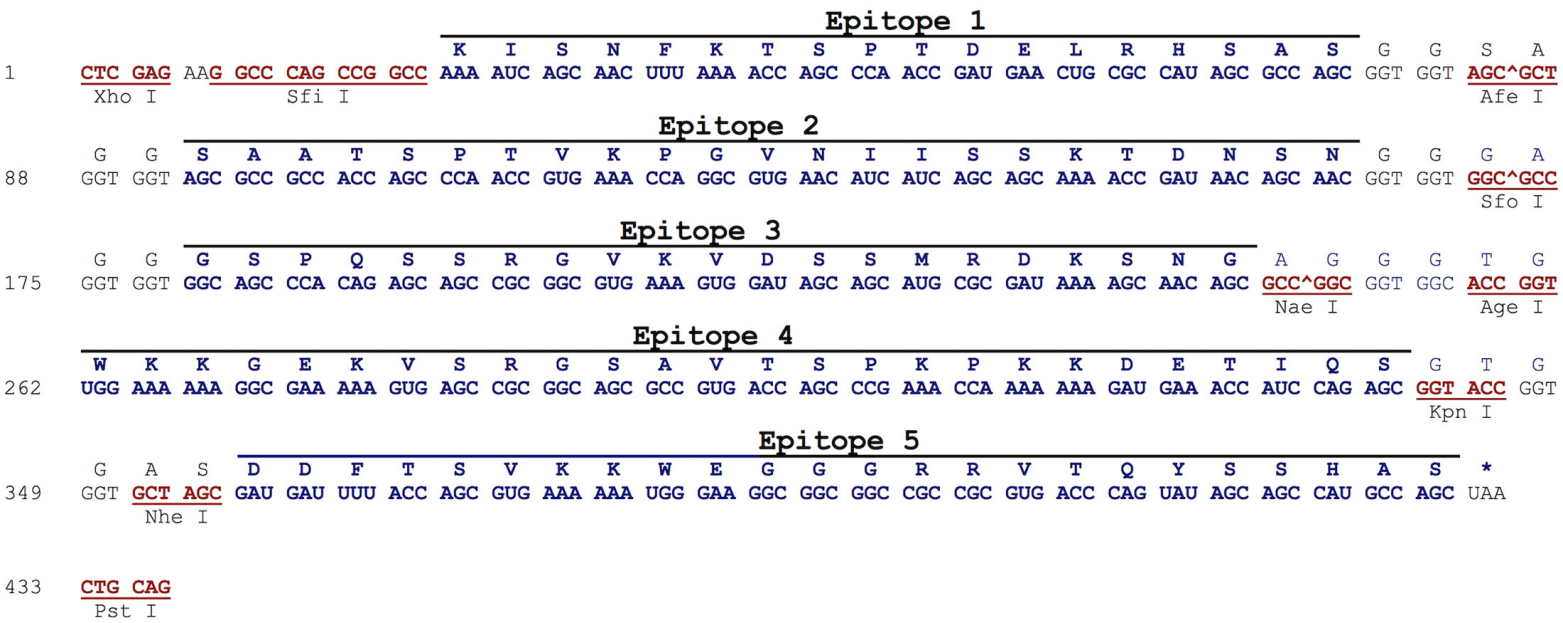
Multiepitope DNA string sequence coding for epitopes detected from the selected 3FTx from *M*. *corallinus*. Cysteine codons were exchanged by serine codons to avoid the formation disulphide bond-mediated protein multimerisation. Epitopes were separated by a six residues linker. Epitopes 1 and 2 are from Ag1, while Epitopes 3, 4 and 5 are from Ags 2, 3 and 4, respectively. Restriction sites (red sequences) were inserted between epitopes to allow further DNA manipulation when required.

**Fig 5:**
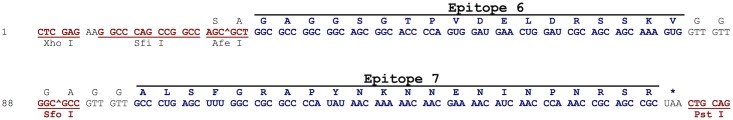
Multiepitope DNA string sequence coding for epitopes detected from the putative PLA_2_ from *M*. *corallinus*. Cysteine codons were exchanged by serine codons to avoid the formation of disulphide bond-mediated protein multimerisation. Epitopes were separated by a six residues linker. Restriction sites (red sequences) were inserted between epitopes to allow further DNA manipulation when required.

Having identified the most reactive peptides from all the five selected neurotoxins, two synthetic multiepitope DNA strings were designed, as previously described, based on the amino acid sequence of those epitopes. One of them, named *3ftx*, codes for all five reactive epitopes associated with the four 3FTx (Fig 4). The other one, named *pla2*, codes for the two reactive epitopes associated with the PLA_2_ toxin (Fig 5). In both cases, cysteine codons were replaced by serine codons to avoid the formation of disulphide bond-mediated protein multimerisation. All epitopes were separated by a six residues linker and codons were optimised for both *Mus musculus* and *Escherichia coli* expression.

### Immunisation assays and venom neutralisation

#### Genetic immunisation with full cDNA sequences of selected antigens

**Fig 6:**
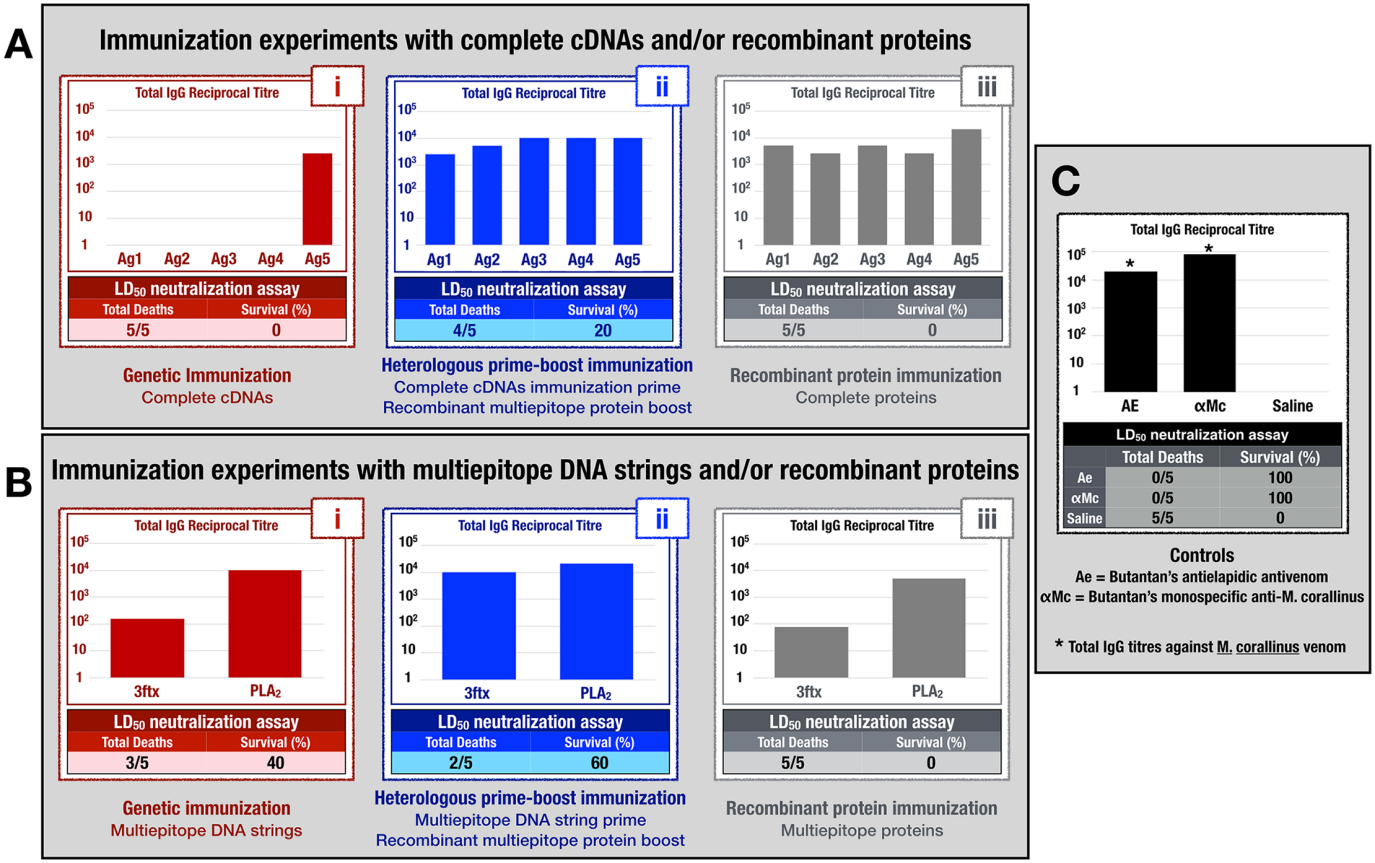
Total IgG antibody titres and neutralising activities of different sera generated through the immunisation experiments performed. (A-i) Total IgG titres and neutralising activity of pooled sera from mice genetically immunised with the complete cDNA coding sequences of all five selected antigens. (A-ii) Total IgG titres and neutralising activity of pooled sera from mice subjected to a heterologous cDNA prime-recombinant multiepitope protein boost immunisation regimen. (A-iii) Total IgG titres and neutralising activity of sera from mice immunised with only the recombinant proteins. (B-i) Total IgG titres and neutralising activity of sera from mice genetically immunised with the multiepitope DNA strings. (B-ii) Total IgG titres and neutralising activity of sera from mice subjected to a heterologous multiepitope DNA prime-recombinant multiepitope protein boost immunisation regimen. (B-iii) Total IgG titres and neutralising activity of sera from mice immunised with only the multiepitope recombinant proteins. (C) ***Positive controls*:** Total IgG titres and neutralising activities for the antielapidic antivenom and for the monospecific anti-*M*. *corallinus* horse antiserum. ***Negative control*:** Total IgG titres and neutralising activities for the physiological saline solution. ELISA assays were performed in duplicate and titres were specified as the last dilution of the sample whose Abs_492nm_ ≥ 0.1. (*) Determination of total IgG titres of either the antielapidic antivenom or the monospecific anti-*M*. *corallinus* horse antivenom, microtitres plates were coated with 1μg of *Micrurus corallinus* venom per well.

As previously described, the complete cDNA sequences of all the five previously selected antigens [22] were cloned into pSECTAG2A vectors and subjected to a genetic immunisation regimen in BALB/c female mice. Here, the observed results showed that the only group of animals that displayed detectable IgG antibody titres was the one immunised with the pSECTAG2A-*pla2* vector, which codes for the putative PLA_2_ toxin (Fig 6A-i). It is worth noting, however, that ELISA microtitre plates were coated with 1μg (*per* well) of recombinant antigens expressed in *E*. *coli* and purified (by IMAC chromatography) under denaturing conditions (8M Urea) from inclusion bodies. After being eluted, these antigens were dialysed against a 3M urea-PBS buffer to avoid protein precipitation. For this reason, we have to consider that this lack of immunoreaction observed could actually be the result of the presence of conformational antibodies that do not bind to incorrect folded antigens. Unfortunately, this was the only way to perform our ELISA assays, since we did not have enough amounts of venom or active toxins. Nonetheless, when these sera were pooled and used for lethal dose assays, no venom neutralisation could be observed (Fig 6A-i), a result that not only correlates with those low IgG anti-3FTx titres but also reinforces the importance of these immunoglobulins for venom neurotoxicity neutralisation.

**Fig 7:**
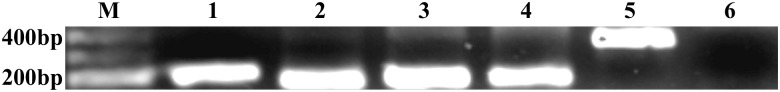
RT-PCR from COS-7 cells transfected with all pSECTAG2A constructions. (M) 1kb Plus DNA ladder. (1) RT-PCR from pSECTAG2A-*ag1* transfected cells. (2) RT-PCR from pSECTAG2A-*ag2* transfected cells. (3) RT-PCR from pSECTAG2A-*ag3* transfected cells. (4) RT-PCR from pSECTAG2A-*ag4* transfected cells. (5) RT-PCR from pSECTAG2A-*ag5* transfected cells. (6) RT-PCR from pSECTAG2A (empty plasmid) transfected cells.

**Fig 8:**
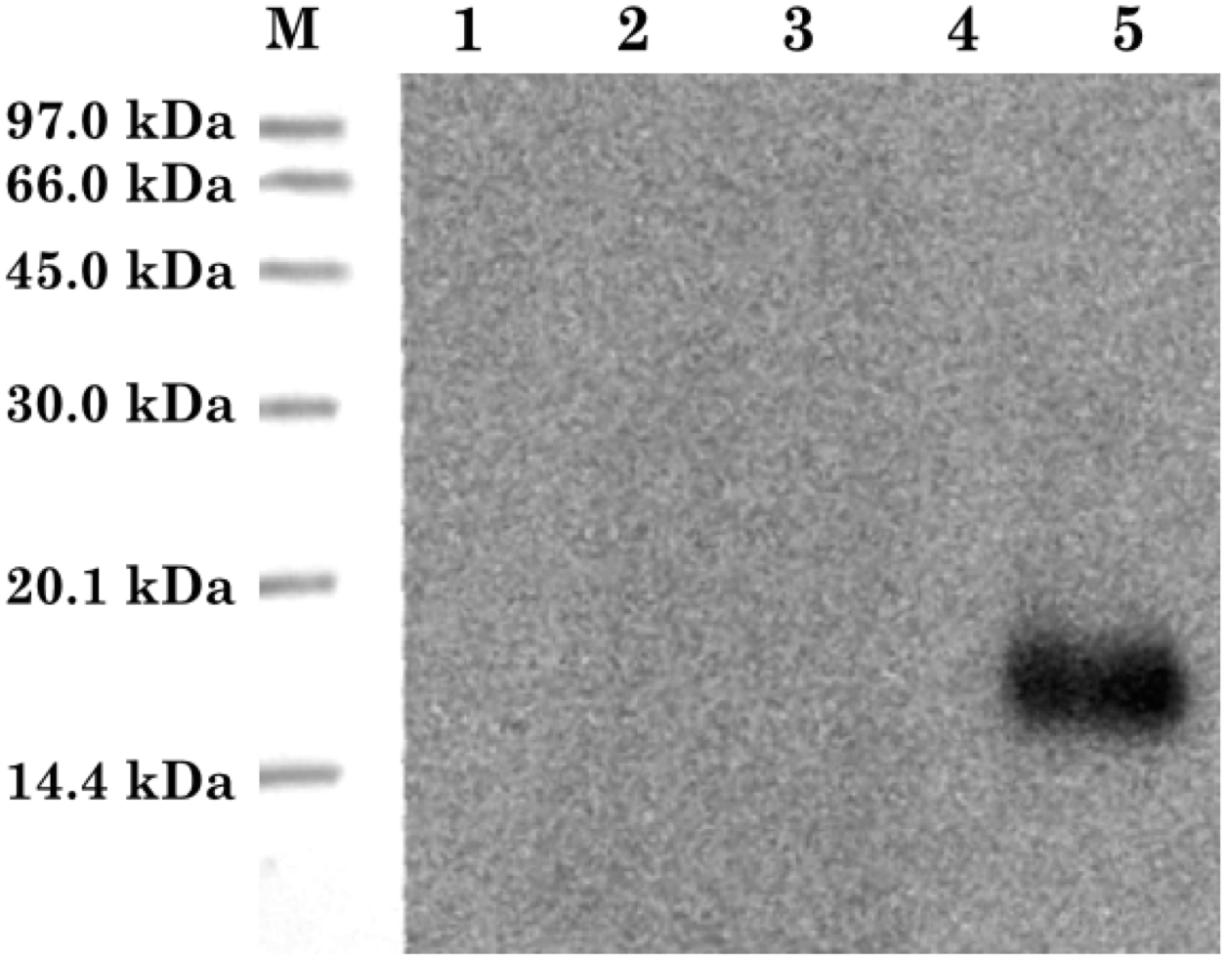
Western-blot analysis of extracts from COS-7 cells transfected with pSECTAG2A constructs. (M) Ponceau-stained low molecular marker (GE Healthcare) transferred to the nitrocellulose membrane. (1) Protein extract from COS-7 cells transfected with pSECTAG2A-*ag1*. (2) Protein extract from COS-7 cells transfected with pSECTAG2A-*ag2*. (3) Protein extract from COS-7 cells transfected with pSECTAG2A-*ag3*. (4) Protein extract from COS-7 cells transfected with pSECTAG2A-*ag4*. (5) Protein extract from COS-7 cells transfected with pSECTAG2A-*ag5*. An anti-*Micrurus corallinus* monospecific horse antiserum was used as primary antibody.

Next, we hypothesised that these low IgG titres could be associated with a problem in RNA transcription and protein translation by host cells, however, when all constructions were transfected into COS-7 cells, mRNAs from all antigens could be detected by *RT*-PCR, indicating that all cDNA sequences were being correctly transcribed (Fig 7). On the other hand, when a Western Blot, immunostained with an anti-*Micrurus corallinus* monospecific antiserum was performed with cell extracts, only the phospholipase A_2_ protein could be detected, indicating that all of the three-fingered toxins were not translated at sufficient quantities by COS-7 cells for being detected (Fig 8), corroborating our suggestion of low protein synthesis by host cells.

#### Heterologous cDNA prime-recombinant multiepitope protein boost immunisation

Considering the fact that even very low concentrations of correctly folded three-finger toxins could sensitise the immune system of genetic immunised animals, we decided to conduct heterologous booster doses with recombinant multiepitope proteins expressed in *Escherichia coli*. For this, we hypothesised that the contact with reactive epitopes, even under denaturing conditions (3M Urea), would elicit an intense response from the already sensitised immune system.

As expected, after receiving the recombinant protein booster doses, the animals not only generated detectable titres of IgG antibodies to all of the 3FTx, but also a substantial increase in the total IgG anti-PLA_2_ titre was observed (Fig 6A-ii). However, when these sera were pooled and used for lethal dose neutralisation assays, no animals survived after venom challenge (Fig 6A-ii).

#### Genetic immunisation with multiepitope DNA strings

Differently from what we observed in those animals that were genetically immunised with total cDNA sequences, the genetic immunisation of mice with both multiepitope DNA strings not only elicited detectable levels of IgG antibodies but also a 40% neutralisation could be observed when pooled sera from these animals were used in lethal dose assays (Fig 6B-i). It is important to notice, here, that the anti-PLA_2_ multiepitope titres were considerably higher than the anti-3FTx multiepitope, ones. This strongly suggests that the putative phospholipase A_2_ from *M*. *corallinus* is, as a matter of fact, much more immunogenic than the four selected 3FTx antigens.

#### Heterologous multiepitope DNA string prime-recombinant multiepitope protein boost immunisation

In a way similar to the previously described heterologous prime-boost immunisation protocol, we decided to conduct recombinant multiepitope protein booster doses to the animals that were primed by genetic immunisation with the multiepitope DNA strings. Again, a much higher immune response could be observed (Fig 6B-ii). And when these sera were pooled and used for lethal dose assays, a much higher neutralisation could be observed (60%), with a total of three, out of five, animals surviving venom injection (Fig 6B-ii).

#### Recombinant protein immunisation

In order to determine if the observed neutralising activities of sera generated during heterologous DNA prime-protein boost regimens could be reproduced without the DNA prime counterpart, we also performed the immunisation of mice by the intraperitoneal injection of purified recombinant proteins, only.

Here, despite the high IgG immunoglobulins titres detected, neither the sera from mice immunised with the recombinant multiepitope proteins nor the sera from mice immunised with the complete recombinant antigens displayed some sort of neutralising activity (Fig 6A-iii and 6B-iii). These low neutralisation results could also be associated with the presence of high concentrations of lipopolysaccharides (LPS) in formulations composed of *E*. *coli*-derived recombinant immunogens, as it is well established that LPS shifts the immune response towards a more Th1-like (cellular) profile [39], which is not ideal for an antiserum development. In antibothropic or anticrotalic sera, the highest neutralising activities observed are due to the IgG(T) immunoglobulin isotype [40, 41], which is not only produced in large amounts by hyperimmunised horses [42, 43] but is also associated with a more Th2-like (humoral) response [44].

Likewise, there are no reasons for us to believe that a different immunological response should be elicited in immunised animals during the development of an antielapidic antiserum. Indeed, the genetic immunisation of mice by GeneGun not only elicits a more Th2-like immune response, but this response is maintained even after subsequent heterologous booster doses of *E*. *coli-*derived recombinant immunogens [45], corroborating the better neutralising results observed in our heterologous DNA prime-recombinant protein boost regimens.

### Final considerations

Although most of the registered cases of snakebite envenomation are due to snakes from the Viperidae family, accidents involving members of the Elapidae family do occur. Additionally, coral snakes, which are the only elapids found in the New World, possess one of the most potent venom found in snakes, which tend to have significant neurotoxicity, inducing peripheral nervous system depression in a way similar to curare poisoning, with muscle paralysis and vasomotor instability. Actually, accidents caused by coral snakes could be very severe or even lethal [3].

Since the early observations from Calmette and Vital Brazil [46], the only acceptable medical treatment for snakebite accidents is the administration of an antiserum generated by horse immunisation with snake venom. Here, for what concerns an antielapidic antivenom, due to a number of factors such as the small size of glands, fossorial habit and very low survival rates in captivity, the production of sufficient amounts of antivenom is jeopardised by the inadequate amount of venom available. In fact, there are already registered cases of patients being intubated and ventilated as a consequence of antivenom shortage in USA, leading to increased morbidity and mortality [11]. Under these circumstances, the development of a new and efficient procedure for coral snake antivenom development, with less reliance upon snake collection and maintenance, would be an important contribution for the treatment of coral snakebite accidents.

In a recent work, the B-cell epitope mapping of *M*. *corallinus* antigens was described and showed promising results when these epitopes were used as peptide antigens [47]. Here, on the other hand, we describe the design and synthesis of two multiepitope DNA strings through the identification of linear B-cell epitopes of five major toxins (four 3FTx and one PLA_2_ [22]) from the venom of *M*. *corallinus*. When these multiepitope DNA strings were used for the genetic immunisation (by GeneGun) of mice, detectable levels of specific antibodies with partial (40%) neutralisation capabilities in lethal dose assays were observed (Fig 6B-i). Furthermore, when these multiepitope DNA strings were used for the expression and purification of recombinant multiepitope proteins, which, in turn, were administered to those previously genetically immunised groups of mice, not only the IgG antibody titres increased but a 60% neutralisation capability was also observed in lethal dose assays (Fig 6B-ii), showing that both multiepitope DNA strings can be used for the generation of neutralising antibodies against *M*. *corallinus* toxins. These results also confirm that transcriptomic studies can provide potential targets for the development of neutralising antibodies and further studies concerning the characterisation of other B-cell epitopes, other formulations and immunisation protocols could help to improve venom neutralisation.

At last, but not least, the fact that a neutralisation of 100% could not be observed does not disqualifies this approach as a promising alternative method for the development of an antielapidic antiserum. As a matter of fact, it is worth noting that all the neutralisation capabilities observed in this work were, as expected, intimately related with the antibody titres. Unfortunately, however, the total volume of sera withdrawn from immunised animals was not sufficient to obtain reliable quantities of purified immunoglobulins, what would be, indeed, an interesting outcome of this work.

### List of accession numbers for genes and proteins mentioned in the text

**GenBank IDs**
AJ344067.1—*Micrurus corallinus* neurotoxin homologue 8 (*nxh8* gene)–mRNA coding for Ag1.
AF197565.1—*Micrurus corallinus* alpha neurotoxin homolog 7 (*nxh7/3/1* gene)–mRNA coding for Ag2.
GQ139600.1—*Micrurus corallinus* MCOR0039C 3FTx precursor–mRNA coding for Ag3.
GQ139603.1—*Micrurus corallinus* MCOR0100C 3FTx precursor–mRNA coding for Ag4.
AY157830.1—*Micrurus corallinus* phospholipase A_2_ –mRNA coding for Ag5.

**Protein Data Bank (PDB) IDs**
3NDS—Crystal structure (1.20Å) of a short neurotoxin from *Naja palida*.
1NOR—NMR structure of neurotoxin II from *Naja naja oxiana*.
2H8U —Crystal structure (2.10Å) of Bucain, a cardiotoxin from the Malayan Krait *Bungarus candidus*.
4IYE—Crystal structure (1.95Å) of green mamba *Dendroaspis angusticeps*, ρ-Da1a toxin.
1YXH—Crystal structure (1.86Å) of a phospholipase A_2_ from *Naja naja sagittifera*.
